# Tonsillotomy versus tonsillectomy on young children: 2 year post surgery follow-up

**DOI:** 10.1186/s40463-014-0026-6

**Published:** 2014-07-27

**Authors:** Elisabeth Ericsson, Jonas Graf, Inger Lundeborg-Hammarstrom, Elisabeth Hultcrantz

**Affiliations:** 1 School of Health and Medical Sciences, Örebro University, Örebro, Sweden; 2 Department of Anesthesia and Intensive Care, Linköping University, Linköping, Sweden; 3 Division of Otorhinolaryngology, Department of Clinical and Experimental Medicine, Linköping University, Linköping, Sweden; 4 Division of Speech and Language Pathology, Department of Clinical and Experimental Medicine, Linköping University, Linköping, Sweden

**Keywords:** Tonsillotomy, Tonsillectomy, Quality of life, Methodology

## Abstract

**Objectives:**

To study the long-term effect of tonsillotomy and tonsillectomy in young children after two years in comparison to the results after six months.

**Method:**

Children, age 4-5 with Sleep Disordered Breathing (SDB) and tonsil hyperplasia, were randomized to TE (32) or TT (35). TT was performed ad modum Hultcrantz with radiofrequency technique (Ellman). An adenoidectomy with cold steel was performed in the same session for 80% of cases. The patients were assessed prior to surgery, at six and 24 months postoperatively. Effects of surgery were evaluated clinically, through questionnaire (general health/snoring/ENT-infections), Quality of Life (QoL), survey of pediatric obstructive sleep apnea with OSA-18, and children’s behavior with the Child Behavior Checklist.

**Results:**

After two years there was still no difference between the groups with respect to snoring and frequency or severity of upper airway infections. Both TT and TE had resulted in large improvement in short and long term QoL and behavior. Three TT-children and one TE child had been re-operated due to recurrence of obstructive problems, the TE-child and one of the TT-children with adenoidectomy and two of the TT-children with tonsillectomy. Three of the TT-children had tonsil tissue protruding slightly out of the tonsil pouch and twelve TE-children had small tonsil remnants within the tonsil pouches, but with no need for surgery.

**Conclusion:**

Younger children have a small risk of symptom-recurrence requiring re-surgery within two years after TT. For the majority, the positive effect on snoring, infections, behavior and quality of life remain and is similar to TE.

**Electronic supplementary material:**

The online version of this article (doi:10.1186/s40463-014-0026-6) contains supplementary material, which is available to authorized users.

## Introduction

At present, the most common indication for tonsil surgery in children is upper airway obstruction causing Sleep Disordered Breathing (SDB) [1]. SDB is a symptom-complex including not only snoring and sleep apnea, but also restless sleep, frequent awakenings, failure to thrive and behavioral disturbances. Oral breathing is often associated with SDB and may cause subsequent bite aberrations [2]. Daytime health related quality of life (HRQL) and level of functioning have been found to be affected by SDB [3]-[7]. Simple snoring without other symptoms of SDB, usually does not qualify a child or an adult for tonsil-surgery.

SDB in children is most commonly caused by a relative hypertrophy of the Waldeyer ring, which usually peaks in size around the age of five [8]-[10]. That is why tonsil surgery due to SDB is especially common in the pre-school age groups [3].

During the past decade, tonsillotomy, or intracapsular tonsillectomy, partial removal of the tonsils, has become accepted as the surgical method for tonsil hyperplasia because it causes less surgical trauma, carries less risk for serious bleedings than total tonsillectomy, and allows for a more rapid recovery [1],[11].

The aim of the present investigation is to study the long-term effect of tonsillotomy and tonsillectomy in young children after two years in comparison to the results after six months and to assess whether the beneficial effects persisted that were observed after six months [4] on snoring, infections, HRQL and behavior.

## Methods

The study was approved by the Human Research Ethics Committee at Linköping University.

### Subjects

Children (4.5–5.5 yrs), who all had tonsil hypertrophy and obstructive problems (SBD), assessed by an ENT-surgeon and been put on the waiting-list for tonsil surgery, had been randomized to either TT(35) or TE(32) [4]. In accordance with Swedish praxis, no sleep studies had been performed on these “otherwise healthy” children, who were neither obese nor had signs of severe OSAS. Sixty-seven children were enrolled, 28 girls and 39 boys, aged 50–65 months (mean age, 56 months; 4.8 years old). Twenty per cent had had one or a few bacterial upper airway infections (tonsillitis) prior to the last three months before surgery. These infections did not exclude them from the study.

Exclusion criteria were recurrent tonsil infections during the last few months, small tonsils, obesity, bleeding disorder or parents not speaking Swedish. No drop outs occurred after enrolment.

Power analysis had been done based on the senior author’s previous study [12], but with more patients included to increase the power and thus make it possible to evaluate group differences in pain and general health.

Randomization had been done from the waiting list (a sequentially numbered list generated by a computer), and families had been informed about the study and the randomization outcome by mail before giving informed consent [4]. Before surgery, the parents had also answered: a disease-specific quality of life questionnaire about general health, snoring, eating difficulties and infections [4],[13],[14], OSA-18 (Obstructive Sleep Apnea-18) [4],[15], and a standardized assessment of child behavior, CBCL (Child Behavior Check List) [4],[16].

TE had been performed on 22 boys and 10 girls and 17 boys and 18 girls underwent TT. 80% (28TT/25TE) underwent adenoidectomy at the same time as primary tonsil surgery and 10% (5TT/1TE) had had an adenoidectomy earlier.

The tonsillectomies were all performed using cold steel. All tonsillotomies were performed ad modum Hultcrantz [13],[17] with the Ellman 4.0 MHz Surgitron Dual Radio wave Unit (Ellman International Oceanside, NY) as follows: The patient was orally intubated and the mouth held open using a David-Myers mouth gag. A neutral electrode under either shoulder was connected to the radio wave unit. Local anesthesia with a vasoconstrictor (0.25% Marcain-adrenaline), was slowly injected into the tonsil tissue on both sides, avoiding leakage through the crypts. To protect the posterior pillars, a gauze strip was placed behind both tonsils, leaving the end of the strip covering the uvula. A small RF-needle was attached to the hand-piece. If necessary, superficial vessels on the tonsillar surface were coagulated using 10% coagulation mode. After switching to 15% cutting mode, an incision was made in the anterior surface of the tonsil, parallel to the anterior pillar. After changing to an Htz tonsil-sling, the incision was widened by a slight medial pull of the tonsil tissue before cutting through the tonsil with a smooth movement (20 per cent output in cut/coagulation mode). If the mouth gag is provided with a suction canal, suction may be connected, thus facilitating steam evacuation. Respecting the first incision plane medial to the tonsil pillars, more tissue may be removed if deemed necessary. An RF needle was used for final hemostasis, avoiding the use of tissue damaging diathermy. Energy levels was adjusted up or down according to cutting effect and steam production [13],[17].

All children participated in the six month follow-up [4]. Two years after surgery, the children were called in for a clinical follow-up, which was not blinded. An ENT-specialist performed a structured interview and examination, which included an estimation of the remaining tonsil tissue inside or outside the pillars in both groups. The interview covered the parents’ evaluation of snoring using a Visual Analogue Scale/VAS (no snoring to severe snoring before, immediately after, and at present two years after surgery). Parents were asked about episodes of upper respiratory infections (URI) with or without treatment with antibiotics, onset of allergies, voice problems/changes, appetite, enuresis and mouth breathing.

The same questionnaires that had been given six months after surgery were administered: the questionnaires about general health, snoring, eating difficulties and infections, OSA-18, and CBCL, were used, with the specific instruction that the same parent as before filled them out. The patients who reported episodes of antibiotic-treated URTI after surgery were further investigated and characterized after their medical charts had been obtained from the treating physician.

*Questionnaire* (*Qu)* included 11 questions comparing the time before and after surgery concerning general health, temper, stamina/energy, concentration, snoring prevalence and snoring loudness, appetite, ENT-infections, antibiotic treatment and satisfaction. The questions were assessed on a five-step Likert scale [4],[14].

The *OSA-18* consists of 18 items grouped into 5 domains: sleep disturbance, physical symptoms emotional distress, daytime function, and caregiver concerns [4],[15]. Items are scored on a 7-point ordinal scale that assesses the frequency of specific symptoms, scored from 1, “none of the time” to 7, “all of the time”. Item responses are summed to produce a total score ranging from 18 to 126. A total score less than 60 suggests minor impact on disease-specific QoL, 60–80, a moderate impact, and greater than 80, a major impact. A mean survey score and individual domain mean scores are calculated. The OSA-18 change scores are calculated by subtracting the follow-up mean survey score and the individual domain mean scores from the baseline mean and individual domain mean scores. Positive values indicate clinical improvement and negative values indicate deterioration. The OSA-18 also provides a direct global rating of SDB-related Health Related Quality of Life (HRQL) via 10-point visual analogue scale with specific semantic anchors.

The CBCL was scored to obtain a total problem score, as well as scores for “Internalizing behavior” (sub scores: Withdrawn, Somatic Complaints, Anxious/Depressed) and “Externalizing behavior” (sub scores: Delinquent Behavior and Aggressive Behavior) [16]. Normative data was available from 1991 for the Swedish population for the version of the instrument used. Each item is scored from 0, “not true” to 2, “very true”/“often true”. The scores from the present study were compared to the normative data for a group of school children 6–15 years old [18]. The instrument consists of two parts: social competence and behavioral/emotional problems. In the present study, only the items from the latter part have been used. Parents completed the same parts of CBCL, as at the time of surgery and at the six month’s assessment.

Statistical analysis was performed with SPSS® Windows version 17.0. Parametric data are expressed as number of cases and mean ± standard deviation (SD). Non-parametric data are expressed in median and inter-quartile range. Non-parametric methods were employed since the variables are at an ordinal level of measurement and the data is not normally distributed (Kolmogorov-Smirnov test). The Wilcoxon signed rank test was conducted on the change scores at 6 months and 2 years. The Mann–Whitney U-test was used for comparison between two subgroups in the questionnaires. Spearman’s rank-correlation coefficient was used for correlation between questions. Differences in CBCL between the population in this study and the comparative normative populations [18] were tested using Student’s *t* test (2-tailed) for normally distributed continuous variables. *P* values <0.05 were considered statistically significant.

## Results

At the two year follow-up, all 67 children answered the questionnaires and 64 children (95.5%) came to the clinical examination.

At the six-month follow-up, no differences in frequency and loudness of snoring or ENT-infections had been noted between the TT and the TE group [4].

At the present two year follow-up, the structured interview showed no difference between the TT and the TE group concerning snoring although three children had been re-operated.

At the ENT examination, one of the 33 TT-children was found to have tonsil tissue slightly outside the tonsil pouch. The parents noted some snoring (VAS 4), but less than before surgery and felt there was no need for re-surgery. 12/32 TE-children had small remnants of tonsil tissues within the tonsil pouches, but none of them reported any significant snoring.

The median value for parental evaluation of severity of snoring before versus two years after surgery with VAS was 8.4 before and 1.3 after TE(n = 32) and 8.5 before and 1.6 after TT(n = 33) (ns).

One TT-child and one TE-child had undergone adenoidectomy due to recurrence of snoring later than the six-month control. Two TT-children (5.9%) were tonsillectomized due to recurrence of snoring after the six-month control, both being of normal weight. One of them had also had two episodes of tonsillitis after 6 months. After re-surgery with TE, this child started to snore again and a new recurrence of adenoid was diagnosed. This time the snoring was alleviated with nasal steroids. A third child with no recurrence of snoring had a re-TT due to severe enuresis and encopresis, which, according to the parents, had been temporarily improved after the first surgery with TT. No positive effect on encopresis was noted after the second operation. This child has since then had continued contact both with the Paediatric clinic and the Child Psychiatric clinic.

No generally increased tendency for upper airway infections was noticed in either group. Antibiotic-treated throat infections were reported by eight TT-children and one TE. The charts of these patients were acquired, showing that three were diagnosed with a positive Rapid Strep Test, the rest (5) had been diagnosed without any objective measures. Two had suffered from recurrent infections preoperatively and one of them had also undergone re-surgery. One child of the five had been treated because of streptococcal infections in the family although asymptomatic himself. One TE-child reported three antibiotic-treated episodes of throat infections after surgery, which was, however, still fewer than he had had before surgery.

Oral breathing was reported in 17/65 children equally in the groups, compared to 40/67 before surgery and 8/65 after six months [19]. In three TT children and two TE children, the oral breathing was only during sleep (ns). No change of voice quality was observed by the parents or the examiner in either group. No child in either group had developed any allergies after surgery.

**Figure 1:**
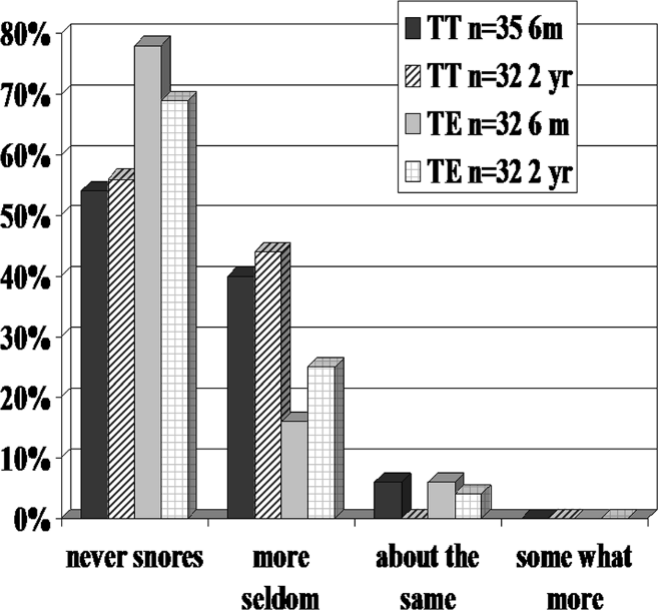
**The frequency of snoring after surgery (six months and two years) in comparison with snoring before surgery (Qu) rated by parents.** The children reoperated with tonsil surgery are excluded in the two year follow-up.

**Figure 2:**
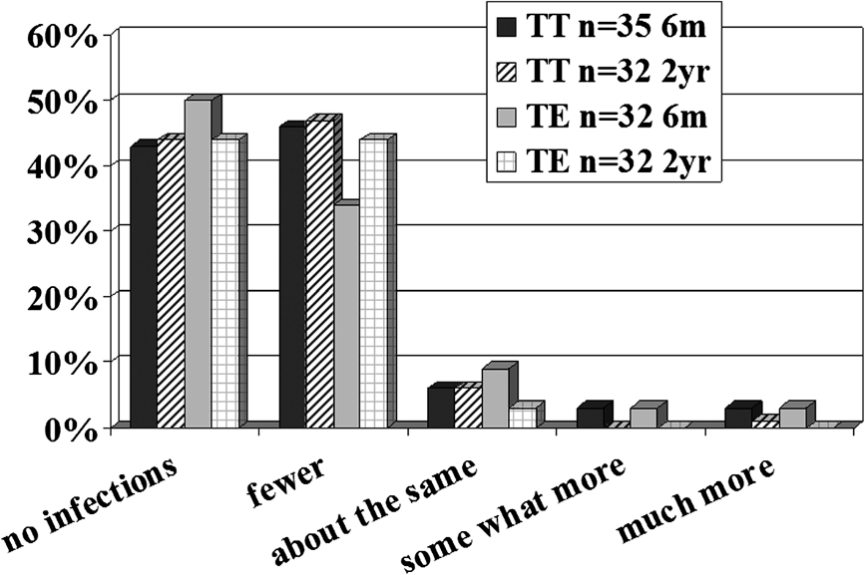
**Assessment of prone to ENT infections children (Qu) were after surgery, six months and two years.** The children reoperated with tonsil surgery are excluded in the two year follow-up.

The questionnaire about general health (Qu) did not show any significant changes between six months and two-years concerning general health, frequency or loudness of snoring (Figure 1) or number of ENT infections (otitis and URI including sore throat, Figure 2).

**Figure 3:**
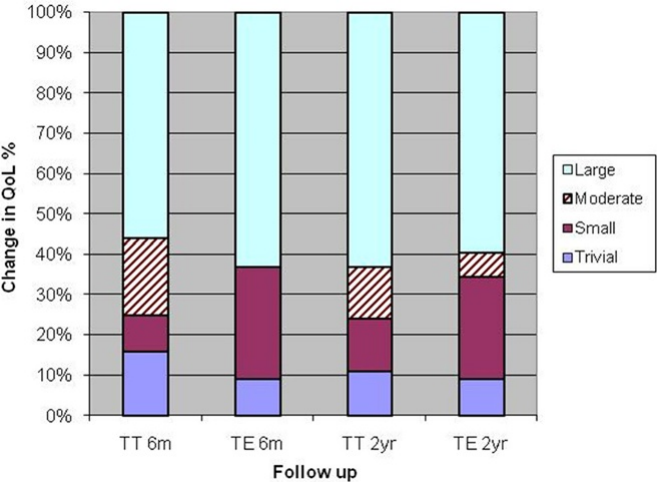
**TT=32/TE=32 Change in disease-specific quality of life 6 months and 2 years after.** Tonsillotomy and Tonsillectomy. The children reoperated with tonsil surgery are excluded in the two year follow-up.

The results from OSA-18 are shown in Table 1 where the preoperative data are compared with the 6-month results and the 2-year follow-up. “Sleep disturbance” and “physical suffering” were the highest rated domains. There was no difference between the TT and TE groups in the improvement of scores after two years, (Table 2 and Figure 3). The total OSA-18 score and each of the domain scores and the visual scale showed large improvement for both tonsillectomy and tonsillotomy after six months (p < 0.0001), an improvement which persisted after two years (see Figure 3). After six months, the “emotional distress” change was moderate and after two years, a major change was noted (Table 2). A fair to good correlation was seen between OSA-18 total score and CBCL total problems preoperatively, and also the postoperative changes in those measures correlated fairly well. After two years there was no difference compared to normative value and the study groups’ for “externalization” and “total problem” (see Table 2) and no differences between the groups.

**Table 1:** **Preoperative responses for the OSA-18 and change scores in TT and TE**

	TT pre	TT change score^b^ at 6 mo	TE pre	TE change score^b^at 6 mo	P-value^c^TT/TE change scores at 6 mo	TT change score^b^pre to 2 yr	TE change score^b^pre to 2 yr	P-value TT/TE change scores pre to 2 yr
	n=35	n=35	n=32	n=32		n=32	n=32	
Total	3.5±1.0	1.8±1,0	3.4±1.0	1.8±1,0	NS	1.8±1,2	1.9±1,4	NS
Sleep disturbance^a^	4.2±1.3	2.7±1.5	3.9±1.4	2.6±1.5	NS	2.9±1.5	2.5±1.3	NS
Physical symptoms^a^	3.9±1.3	1.9±1.7	3.8±1.4	2.1±1.6	NS	2.1±1.5	2.0±1.4	NS
Emotional distress^a^	3.2±1.7	1.0±1.4	3.1±1.4	1.1±1.5	NS	1.6±2.1	1.7±1.8	NS
Daytime function^a^	3.1±1.4	1.2±1.5	3.3±1.3	1.7±1.2	NS	1.5±1.3	1.3±1.3	NS
Caregiver concerns^a^	3.0±1.6	1.6±1.5	2.8±1.4	1.5±1.3	NS	1.5±1.5	1.5±1.3	NS

^a^mean ± standard deviation (SD; ^b^Change score=The follow-up mean survey score and the individual domain scores subtracting from the baseline mean and individual domain mean scores ^c^Mann-Whitney U-Test. (Change score<0.5=trivial change; 0.5 to 0.9= small change; 1.0 to 1.4 =moderate change; and ≥1.5= large change). The children reoperated with tonsil surgery are excluded in the two year follow-up.

**Table 2:** **Child Behavior Checklist before and after surgery, TE resp. TT and compared with normal range**

	TT	TE	P-value TT CBCL/normal range^a,c^	P-value TE CBCL/normal range^a,c^	P-value TE/TT CBCL
*Before surgery*	n=35	n=32			
Internalization^b^	5.8±4.6	4.2±3.6	<0.01	NS	NS
Externalization^b^	9.8±7.0	7.8±6.1	NS	NS	NS
Total problems^b^	25.6±19.1	20.9±12.4	<0.001	<0.01	NS
*Six months after surgery*	n=35	n=32			
Internalization^b^	3.7±5.4	2.4±2.7	NS	<0.05	NS
Externalization^b^	7.6±6.7	6.3±4.5	<0.05	NS	NS
Total problems^b^	19.5±18.4	13.5±9.8	<0.05	NS	NS
*Two years after surgery*	n=32	n=32			
Internalization^b^	3.1±3.9	2.9±5.8	NS	<0.05	NS
Externalization^b^	5.6±6.0	5.6±6.9	NS	NS	NS
Total problems^b^	13.9±12.9	13.6±21.7	NS	NS	NS

NS= not significant. ^a^Normal range. Swedish population [18]^b^Mean ± SD ^c^Mann-Whitney U-test. The children reoperated with tonsil surgery are excluded in the two year follow-up.

After two years there was no difference compared to normative value and the study groups’ for “externalization” and “total problem” (see Table 2) and no differences between the groups

## Discussion

Concerns about re-growth and recurrence of obstructive problems have been raised after partial removal of tonsils, especially with respect to younger children with a naturally rapid growth of the lymphatic tissue in the Waldeyer ring and at the same time narrow dimensions of the upper airway [8],[10]. The present study demonstrates equally good long-term results on recurrence of SDB for RF-tonsillotomy as for traditional tonsillectomy in a young group of patients.

There is however a certain risk of re-growth of tonsil tissue and recurrence of obstructive problems. How great that risk is, is not possible to evaluate with the power of the present study although recurrence rates of six to seven per cent among very young children [20],[21] and around three per cent or lower [1],[22]-[25] among older children have been noted in other studies. Large material, such as those in register studies, are needed for these kinds of analyses. In the present study, a couple of the TT children had undergone further tonsil surgery within two years due to recurrence of snoring and one child in each group had had another adenoidectomy.

Tonsillotomy methods do vary considerably, with some techniques aiming at removing as much tonsil tissue as possible, leaving only a thin layer as a “biological dressing”. In the present study, we aimed at only removing the obstructing tonsil tissue protruding medially of the pillars, thereby reducing the risk of bleeding, and also resulting in less pain as well as shorter operating time. The technique used in the present study (ad modum Hultcrantz) has the advantage that the risk of damage to big blood vessels is minimal and when compared to laser and diathermy, TTRF also has the advantage that less lateral heat spreads to the underlying tissues (reducing the risk for late bleedings).

Due to the study plan, TE was performed after recurrence of snoring (in two children), but re-TT would probably have been just as effective, and in our clinical praxis, re-TT is often the parent’s choice, although tonsillectomy could be advocated to avoid yet another setback [21].

Apart from the fear of re-growth, infections have been considered a risk after tonsillotomy, but no significant difference between the TT and TE group was observed in the frequency of upper airway infections. This is consistent with earlier findings [14]. One explanation could be that a high proportions of TE-children also were shown to have remnants of tonsil tissue in the tonsil pouches, which might have equalized the groups with respect to immunological defense. Most authors and clinicians recommend TE as method of choice in cases with recurrent infections, although very few studies have addressed throat infections after tonsil surgery. In the present study, more TT-children had been treated with antibiotics for throat infections than among the TE-children. However, most of them were not diagnosed objectively. Our previous study [14], including older children, showed an equal rate of infections, 12%, in both the TT and TE group. A possible confounding factor concerning antibiotic treatment is that a physician could be more prone to prescribe antibiotics to a child with parts of the tonsils left than to a patient thought to have no tonsils left at all. Contrary to most other studies, which exclude children with recurrent tonsillitis, we suggest that TT can be performed also on children with obstructive symptoms and a “normal” rate of throat infections, a number that would not per se qualify for tonsillectomy. Since we could not require that the parents should seek an ENT specialist when their child came down with a sore throat, it was difficult to get a thorough assessment of a diagnosed tonsillitis; the family doctor did not always take a bacterial swab or motivated the rationale for antibiotic treatment.

Several children continued to breathe through their mouths after surgery despite showing no objective re-growth of the tonsils; the number actually being larger after two years than after six months. This reflects the fact that habitual mouth breathing is a sign of an oro-motor disturbance that can be very difficult to change and may result in further negative impact on maxillary growth. One reason for the difficulties in converting the breathing mode could be the individual/genetic oro-facial shape with a “narrow” maxilla, not allowing free nasal breathing even after surgery of the tonsils and adenoid [26]. To achieve maximum effect from surgery and avoid the risk of further cranio-facial aberrations, a certain emphasis on behavioral training is recommended if a child does not convert spontaneously to nasal breathing after surgery. Surgery can thus be viewed as a step in the treatment. Further orthodontic evaluation is vital if mouth-breathing is not resolved. This in turn can lead to the requirement for maxillary widening [26].

Major improvements in disease-specific and global HRQL as well as in behavioral parameters were noted among all operated children. A weakness in the present study is that we made no comparison with healthy children without SDB or children with SDB who did not undergo tonsil-surgery. Stewart et al. [27] evaluated HRQL for otherwise healthy children with and without OSA and SDB and found improvements in both sleep-study data and HRQL after TE. However the association between sleep-study findings and HRQL was only moderate.

The children in the present study were “otherwise healthy” without any severe obesity, craniofacial aberration or other diseases, so sleep-studies had not been regarded as necessary [28].

Improvements in scores on both the OSA-18 instrument and CBCL noted at the six-month follow-up visit persisted at the two-year follow-up. These improvements in behavior and HRQL could be result of the surgery, but also might be the result of the child’s normal development, which in turn might not have occurred if the SDB had persisted or returned.

There is a relationship between SDB and behavioral problem [3],[7] and a prominent improvement in behavior, cognitive functions and HRQL after tonsil surgery has been noted [7],[27]. Forty per cent more boys than girls were operated. Abramson et al., recently demonstrated that there are no radiographic gender-related differences in children’s airway size [8]. Is tonsillar hypertrophy and SDB more common among 4–5 year old boys than girls? An Icelandic study [29], implies the opposite, with a maximum SDB prevalence among boys at age 2.5 and among girls at age 5. The trend that more boys are operated is clear [21],[23],[30] but not completely consistent in the literature, ranging from twice as many boys [23] to an equal number being operated due to SDB, but with twice as many done due to recurrent infections [30]. The reasons for these differences remain unclear, but the rates of surgery might not reflect actual prevalence of SDB, but rather other, gender attitudes. Parents might be more prone to regard their sons as “tougher” than their daughters and therefore are more willing to let them go through a surgical procedure.

Several surgeons were involved in the study, but no calibration of surgical methods was performed. There is risk for a slide in surgical methods towards own preferences and tonsil surgery is performed in several slightly different manners. Fewer, “calibrated” surgeons, preferably just one, would have ensured less “surgeon-bias”. The surgeons attempted to calibrate their view of tonsil size in an attempt to standardize this part of the study. Records of differences in tonsil size before surgery should have been made, providing an opportunity to stratify the material, for example using the Brodsky-scale rating tonsil size and percentage of the obstruction from one to four [10].

The role of bias is not discussed in most other studies on tonsil surgery, but may occur in several different forms. A recall bias is probable to some extent, since the parents of the patients were asked in retrospect to mark the extent of the child’s snoring on a VAS pre-operatively, soon after surgery, and two years after surgery. The present study was not blinded, which introduces another bias due to expectations. A blinded observer in the two-year follow up would have added strength to the observations and eliminated some observer’s bias. Mink et al. [31] suggest that the family and assessors of outcome should be blinded, as should the surgeon until immediately before surgery.

We strongly believe that most parents would object to having their children undergo surgery without knowing the character of the procedure and that it would be unethical, although the actual difference between the operations may be seen as slight in a layman’s eyes. Quite probably, it would also have affected the drop-out ratio negatively. A surgeon would also object, since the possibility to affect the method of surgery would be eliminated. However, since tonsillotomy has gained increasing popularity among ENT-doctors, more parents know about the method and its possible benefits. Thus, it would have been interesting to perform a single-blinded study to avoid the risk of response bias and observer bias from overly positive expectations from the parents in the TT-group, and perhaps correspondingly negative expectations in the TE-group.

Internal validity also has to be discussed: Did the children stop snoring due to surgery or due to general growth and maturation? We assume that the general growth of children in this age group should provide a “self-healing” effect. In a previous study with children 5–15 years of age [17], a number of included children recovered spontaneously from obstructive sleep disorder and were subsequently excluded and removed from the waiting list.

Pre-school children with tonsillar hyperplasia do however seem to continue to have large tonsils throughout their childhood, as implied by Kaditis et al. [10]. Also, in a non-longitudinal study, Löfstrand-Tideström and Hultcrantz, found that a child who snored at age 4, had a six times greater risk for snoring at age 12 than a child who did not snore at age 4. This was regardless of whether surgery had been performed or not [26].

## Conclusion

Tonsillotomy with RF for children between 4–6 years of age with tonsil hyperplasia comes with/results in a small risk of recurrence within two years. This has to be weighed against the lessened risk for severe pain and dangerous bleedings. The long-term good effect on snoring, infections, behavior and quality of life is similar to that of tonsillectomy.

## Authors’ original submitted files for images

Below are the links to the authors’ original submitted files for images. Authors’ original file for figure 1 Authors’ original file for figure 2 Authors’ original file for figure 3 Authors’ original file for figure 4
