# Supplementary figures and images for: Tonsillotomy versus tonsillectomy on young children: 2 year post surgery follow-up
Source: J Otolaryngol Head Neck Surg. 2014 Jul 27;43:26. doi: 10.1186/s40463-014-0026-6 (PMC6389138; doi:10.1186/s40463-014-0026-6)

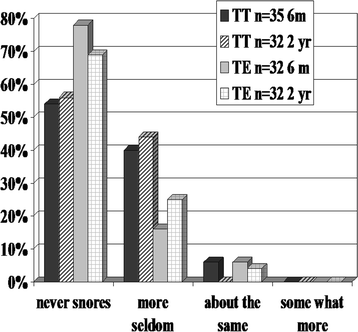

Supplement: Supplementary file 1 — Authors’ original file for figure 1 [file 40463_2014_26_MOESM1_ESM.gif]

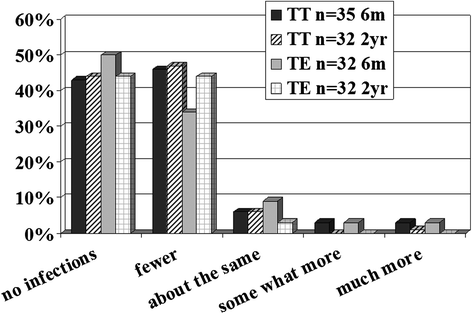

Supplement: Supplementary file 2 — Authors’ original file for figure 2 [file 40463_2014_26_MOESM2_ESM.gif]

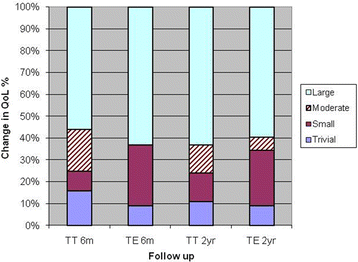

Supplement: Supplementary file 3 — Authors’ original file for figure 3 [file 40463_2014_26_MOESM3_ESM.gif]
