# Supplementary material for: Tonsillotomy versus tonsillectomy on young children: 2 year post surgery follow-up
Source: J Otolaryngol Head Neck Surg. 2014 Jul 27;43:26. doi: 10.1186/s40463-014-0026-6 (PMC6389138; doi:10.1186/s40463-014-0026-6)
Supplement: Supplementary file 4 — Authors’ original file for figure 4 [file 40463_2014_26_MOESM4_ESM.doc]

## Legends

Figure 1 The frequency of snoring after surgery (six months and two years) in comparison with snoring before surgery (Qu) rated by parents. The children reoperated with tonsil surgery are excluded in the two year follow-up.

Figure 2 Assessment of prone to ENT infections children (Qu) were after surgery, six months and two years The children reoperated with tonsil surgery are excluded in the two year follow-up.

Figure3. TT=32/TE=32 Change in disease-specific quality of life 6 months and 2 years after Tonsillotomy and Tonsillectomy. The children reoperated with tonsil surgery are excluded in the two year follow-up
